# Human epidermal growth factor receptor 2 and proliferation Ki-67 biomarkers using a tissue microarray to refine the histopathological subtyping of hydatidiform moles: Limitations and prognostic value

**DOI:** 10.1016/j.clinsp.2025.100766

**Published:** 2025-09-20

**Authors:** Consuelo Lozoya López, Nathália Silva Carlos Oliveira, Fabiana Resende Rodrigues, Ana Luisa Figueira Gouvêa, Karin Soares Gonçalves Cunha, Ana Carolina Brito, Antonio Braga, Vânia Gloria Silami Lopes

**Affiliations:** a Department of Pathology, Faculdade de Medicina, Universidade Federal Fluminense, Hospital Universitário Antonio Pedro (HUAP-UFF), Niterói, RJ, Brazil; b Postgraduate Program in Pathology, Faculdade de Medicina, Universidade Federal Fluminense, Hospital Universitário Antonio Pedro (HUAP-UFF), Niterói, RJ, Brazil; c Department of Maternal and Child Medicine, Universidade Federal Fluminense (UFF), Niterói, RJ, Brazil; d Department of Obstetrics and Gynecology, Universidade Federal Rio de Janeiro (UFF), Rio de Janeiro, RJ, Brazil; e University of Vassouras, Postgraduate Program in Applied Health Sciences, Vassouras, RJ, Brazil

**Keywords:** Hydatidiform mole, Predictive and diagnostic markers, Immunohistochemistry, Tissue Microarray

## Abstract

**Introduction:**

The morphology-based differential diagnosis of Hydatidiform Mole (HM) of the Complete (CHM) and Partial (PHM) types is challenging because of earlier diagnosis of HM owing to the universal application of ultrasound during antenatal care. HMs may present with amplified oncogenes or other gene mutations, resulting in recurrent or neoplastic disease. The cell proliferation markers Ki-67 and HER2 may contribute to the final HM subtype and prognosis. Much is known about the basic mechanisms of HM development; however, other molecular diagnostic and predictive markers need to be investigated.

**Methods:**

This was an ambispective anatomopathological study of 108 HMs cases. A tissue microarray was used for Ki-67 or HER2 immunostaining analysis. Associations between immunomarker scores and postmolar Gestational Trophoblastic Neoplasia (GTN) were analyzed via Fisher’s exact and linear-by-linear association tests.

**Results:**

A statistically significant trend toward increased Ki-67 immunostaining in CHM samples was observed. Seventeen HM patients developed GTN, of whom 6 (35 %) had a Ki-67 score of 3+ and 9 (53 %) had Ki-67 score of 2+. Two (12 %) HM patients had a HER2 score of 3+, and 4 (23 %) HM patients had a HER2 score of 2+, of whom 2 (12 %) patients exhibited oncogene amplification by FISH HER2.

**Discussion:**

Ki-67 and HER2 markers may be useful for the diagnosis and prediction of HM development, providing alternative targeted therapies. This study needs to be interpreted with caution due to its small sample size, high sample exclusion rate, and the absence of significant associations between biomarkers and clinical outcomes.

## Highlights

• Ki-67 and HER2 serve as biomarkers to differentiate CHM from PHM for better prognosis.
• Increased Ki-67 levels in CHM suggest association with disease aggression and prognosis.
• HER2 2+ tumors show amplification, indicating potential for targeted therapies.
• Tissue microarray efficiently analyzes multiple samples, enhancing cost-effectiveness.
• Notable trends in Ki-67 and HER2 suggest utility in prognosis, despite no strong stats.

## Introduction

Hydatidiform Mole (HM) is a genetic anomaly of pregnancy that represents the benign form of Gestational Trophoblastic Disease (GTD), is associated with medical complications such as hemorrhage, hyperemesis, and early preeclampsia, and constitutes a risk factor for GTN development. HM presents itself in two distinct clinical, histopathological, and cytogenetic forms: Complete Hydatidiform Mole (CHM) and Partial Hydatidiform Mole (PHM). The development of postmolar GTN is 4–20 times greater in cases of CHM than in those of PHM.^1^, ^2^, ^3^ The duration of post-molar follow-up is approximately 6 months after remission in cases of CHM, and the time of discharge can be brought forward to 30 days after normalization of hCG in cases of PHM; therefore, differentiating these two conditions is essential.^4^ Strategies for the differential diagnosis of CHM and PHM have been reported in the literature.^5^, ^6^, ^7^ Some studies have evaluated ploidy, immunostaining markers, and genotyping for classifying the subtypes of HM.^1^^,^^8^^,^^9^ Owing to the earlier diagnosis of molar pregnancy due to the universal use of ultrasound in prenatal care, the differential diagnosis of these subtypes can be difficult, especially in recently formed moles.

Because of the morphologic complexity, especially in these cases, the previous study demonstrated three combined strategies (histopathology, analysis of p57 expression, and HER2 FISH positivity) for improving the differential diagnosis of the two molar subtypes, which would increase the ability to predict neoplastic outcomes. The authors also applied the Tissue Microarray (TMA) technique to 108 samples in a single paraffin block. This approach reduces costs (materials and personnel) and time, in addition to enabling the analysis of different cases under the same technical conditions on a single slide.^10^

In the present work, the authors sought to complement previous research by analyzing Ki-67 and HER2 cell proliferation markers in the same 108 samples using the TMA. The Ki-67 marker is a protein encoded by the MKI-67 gene, which is located on chromosome 10q26.2; this protein is expressed in all phases of the cell cycle and has been proven to be a biomarker of tumor aggressiveness and a predictor of neoplastic evolution.^11^ Semiquantitative analysis revealed that the expression of the Ki-67 protein in cytotrophoblastic and syncytiotrophoblastic cells was greater in patients with gestational trophoblastic neoplasia than in patients with uneventful molar pregnancies.^12^

Owing to the reported importance of KI-67 protein expression, HER2/cerb2, another cell proliferation biomarker, which is a transmembrane protein that is a member of the EGFR receptor family derived from a proto-oncogene located on chromosome 17q21.1, was analyzed. Amplification of the oncogene has been reported in several epithelial neoplasms and is associated with several important processes involved in carcinogenesis.^13^ Compared with genotyping studies, which are much more expensive and therefore less accessible in pathology laboratories, analyses of HER2 expression could aid in refining the diagnosis of HM. Additionally, these immunostaining methods are important for developing prognostic approaches and targeted therapies in studies of breast cancer, gastric cancer, ovarian cancer, lung cancer, and even sarcomas.^14^, ^15^, ^16^, ^17^ Therefore, this study was conducted to verify whether this approach would generate more robust results not only in the final diagnosis of MHC or MHP but also in the investigation of molecular predictive markers for the disease and in the consequent search for targeted therapies.

## Materials and methods

### Ethics approval and consent to participate

This study was approved by the Research Ethics Committee of Fluminense Federal University Research (CAAE 64,840,914.9.00005243). This research standard is defined in Resolution National Health Commission (CNS) n° 466 of 2012 and in Operational Standard n° 001 of 2013 of the CNS. All patients signed a free and informed statement (consent to participate declaration).

### Study design

This was an ambispective anatomopathological study of 108 products of conception diagnosed as HMs retrieved from the surgical pathology files of the GTD Reference Center at Hospital Universitario Antonio Pedro (Universidade Federal Fluminense, Brazil) from 2002 to 2017. These 108 cases were included in the previous study.^10^ The cases were diagnosed as CHM, PHM, or inconclusive via a set of three techniques: histopathology, immunohistochemistry for p57 to detect the maternal genome, and FISH—HER2 to detect ploidy and oncogene amplification in the samples. In this study, the authors also used the same TMA to determine the Ki-67 and HER2 immunostaining scores.

Clinical data and disease outcomes were obtained from medical records. The clinical diagnosis of GTN followed the technical criteria of the ‘Federation Internationale de Gynecologie et Obstétrique’ (FIGO2021).^18^

### Tissue microarray

A hollow needle was used to remove a 1.19 mm diameter tissue core from a paraffin donor block from each patient, including areas in which atypia and trophoblast epithelial hyperplasia were detected on the respective histopathological slide. The 108 cylindrical tissue fragments were inserted into a paraffin receiving block utilizing a matrix pattern with precise spacing.^19^ As positive controls for IHC and for spatial orientation of the fragments in the block, the authors utilized tissue cylinders of the cerebellum, intestine, skin, breast, testis, tonsil, and kidney tissue that were included in this block. Sections from this block were subjected to Ki-67 and HER2 immunostaining.

### Ki-67 and HER2 immunohistochemistry

Four-micrometer-thick sections from the formalin-fixed TMA paraffin-embedded blocks were deparaffinized in xylene and alcohol, washed, and rehydrated in distilled water. Endogenous peroxidase activity was quenched with 3 % hydrogen peroxidase solution, and antigen retrieval was performed by placing the samples in 0.01 M citrate buffer (pH 7.0) with 0.1 % Tween-20 for 40 minutes at 98 °C. The sections were cooled for 20 minutes and immersed in 1 mol/L EDTA (pH 9.0) for 40 minutes at 98 °C. Next, the sections were immersed in 1 mg/mL protease XXIV (Sigma-Aldrich. St. Louis, MO, USA) in PBS for 60 minutes at room temperature and washed in water and PBS. Then, the slides were immersed in 5 % goat serum for 20 minutes at room temperature (to block nonspecific reactivity). A mouse monoclonal primary antibody against the human Ki-67 protein (clone MIB 1; mouse; Biogenex Laboratories, CA, USA; 1:100 dilution) or HER2 protein (clone CB11; rabbit; Leica Biosystems, IL, USA; 1/200 dilution) was applied to the samples overnight. A peroxidase-labeled anti-mouse immunoglobulin secondary antibody conjugated with amino acid polymer (Envision system DAKO® Corporation, California, USA) was applied for 60 minutes at room temperature. The sections were rinsed in PBS, treated with 3,3′-diaminobenzidine solution, and counterstained with hematoxylin. The IHC-stained slides were scanned via Aperio CS™ (Leica Biosystems®, USA) at × 40 magnification, and the analyses were performed via ImageScope® software v12.4.0543.

Ki-67 protein expression was semi-quantitatively evaluated by a pathologist who was not involved in this research, was experienced in the semiquantitative analysis of the Ki-67 marker, and was blinded to the course of molar pregnancy follow-up.

The criteria for including the sample in the analysis were the integrity and representation of the area of interest, which should contain at least 50 trophoblastic cells. For each sample, the nuclei of trophoblastic cells present in a field of view under 40 × magnification were analyzed. Samples that did not contain representative molar tissue, those that were predominantly necrotic, those that showed partial loss of the fragment during histological processing, and those that contained only gestational-pattern endometria were defined as non-valuable or inconclusive. The amygdala fragment included in the TMA served as a positive control.

**Fig. 1:**
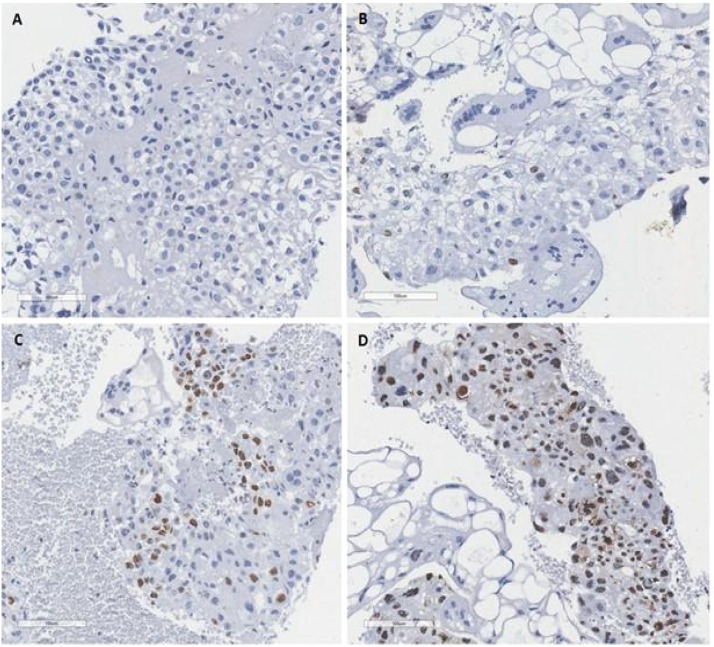
Ki-67 immunohistochemical pattern in hydatidiform moles. (A) Score of 0 (no stained with the brown chromogen in the nuclei of the trophoblastic cells); (B) Score of 1+ (less than 10 % of nuclei positive staining with the brown chromogen in the trophoblastic cells); (C) Score of 2+ (10 % to 50 % of nuclei positive staining with the brown chromogen in the trophoblastic cells); (D) Score of 3+ (more than 50 % of nuclei positive staining with the brown chromogen in the trophoblastic cells). The ki67 is an indicator of proliferative activity of the tumor. Is a nuclear antigen detected by the ki67 antibody in the immunohistochemical study and can be a prognostic and a predictor of treatment response.

Positivity was determined according to the quantification of the stained nuclei of all trophoblastic cells in the sample, and the results were grouped according to the reported study^12^ (Fig. 1). Thus, the authors consider score of 0 (no stained nuclei); score of 1+ (less than 10 % of nuclei positive); score of 2+ (10 % to 50 % of nuclei positive); score of 3+ (more than 50 % of nuclei positive).

**Fig. 2:**
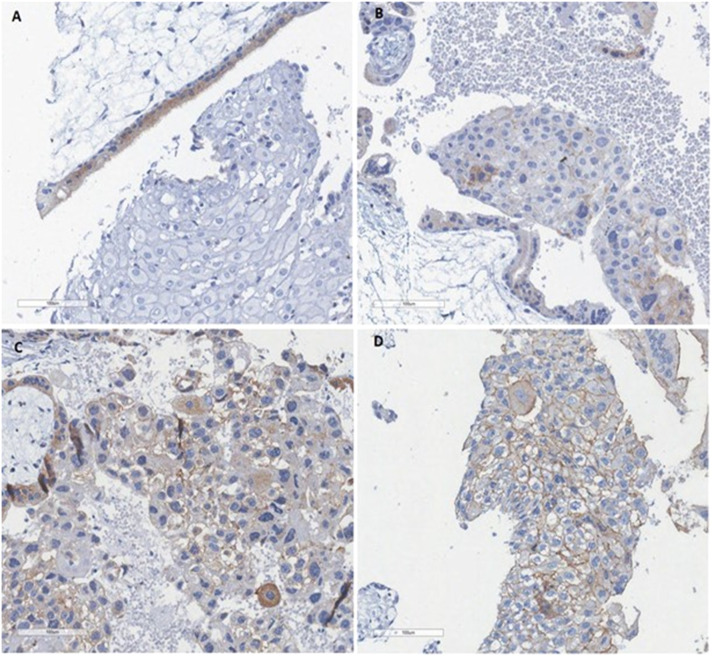
HER2 immunohistochemical pattern in Mole. (A) Score 0 (no detectable membrane labeling with the brown chromogen in the trophoblastic cells); (B) Score + (observation of weak staining in part of the cell membrane in more than 10 % of trophoblastic cells); (C) Score ++ (observation of complete membrane staining of weak to moderate intensity in more than 10 % of trophoblastic cells); (D) Score +++ (strong full membrane staining in more than 10 % of trophoblastic cells). The score +++ means a possible amplification of HER2 and can predict patient prognosis and indicate the possibility of targeted HER2 therapy as an alternative.

Similar to the analysis, HER2 analysis was also carried out by a pathologist who was not a participant in this research, was experienced in the routine semiquantitative analysis of the HER2 marker, and was blinded to the course of molar pregnancy follow-up. HER2 protein expression in the cytoplasmic membrane was evaluated semi-quantitatively in trophoblastic cells. The analysis also included samples with adequate integrity and representation of the area of interest, containing at least 50 trophoblastic cells, within a field of view under 40 × magnification, with subjective quantification of the staining intensity and the proportion of stained trophoblastic cells. The analysis method follows the model already standardized for breast carcinomas.^20^ The exclusion criteria included samples without sufficient representation of molar tissue, samples with an extensive area of necrosis, samples with loss of the fragment during histological processing, and samples in which only the decidua or endometrium was present. The positive controls for the reaction were two fragments of breast carcinoma tissue with a HER2 score of 3. The score for each sample was determined according to a previously reported method^13^ (Fig. 2): score 0 (no detectable membrane labeling with the brown chromogen in the trophoblastic cells); score + (observation of weak staining in part of the cell membrane in more than 10 % of trophoblastic cells); score ++ (observation of complete membrane staining of weak to moderate intensity in more than 10 % of trophoblastic cells); score +++ (strong full membrane staining in more than 10 % of trophoblastic cells).

### Exclusion criteria

Data classified as “not evaluable” during IHC for Ki-67 and HER2, data classified as “inconclusive” at the time of final diagnosis, and data classified as “no record” due to the uncertainty of the “presence or absence of neoplasia” were discarded, as it was not possible to evaluate the association of their status with each of the markers used.

### Statistical analysis

The possible associations between the results obtained for “Final Diagnosis” and “Presence of Neoplasia” and the results obtained for the Ki-67 and HER2 markers were analyzed via 2 × 4 contingency tables. Fisher’s exact test was used to evaluate evidence of an association of each of the binary variables for the final diagnosis and the presence or absence of neoplasia with the variables for the four categories (scores) of marker results.

Assessment of the existence of a trend between the variables for the “Final Diagnosis” of CHM, PHM, or GTN and the ordinal variables for the results obtained for the Ki-67 and HER2 markers was carried out via the linear-by-linear association test, which uses an ordinal chi-square test to identify variation trends. The adjusted residuals of the cells in the contingency table made it possible to identify which categories of variables were responsible for which associations. Adjusted values greater than or equal to 2 were considered to indicate evidence for an association between categories. Tests whose descriptive levels (p-values) did not exceed the significance level α = 0.05 were considered to indicate statistical significance. Data analysis was performed via PASW version 18 (SPSS, Chicago, IL, USA).

## Results

### Patient profiles

The authors evaluated a population of 102 patients, 6 of whom had duplicated samples, for a total of 108 cases. These six patients had repeated episodes of abortion.

Three of the six patients had 2 recurrent moles included in the study. Two had three miscarriages, two of which were included in the present research, and one had six abortions, two of which were included in this study sample. The authors recruited patients whose samples were viable for analysis in the TMA, patients whose final diagnosis was acquired by the previous study with histopathological criteria, p57, and FishHer2 for ploidy, and those whose results were ‘inconclusive’ at the time of final diagnosis, and those whose data in medical records were insufficient. As a result, 78 participants in the Ki-67 TMA were studied, and 77 participants in the HER2 TMA were evaluated. In the Ki-67 TMA, 57 of the 78 participants were CHM patients, and 21 were PHM patients. In the HER2 TMA of 77 participants, 55 were CHM patients, and 22 were PHM participants. Sixty-nine Ki-67 HM patients and sixty-seven HER2 HM patients were prospectively followed up.

### Semiquantitative analysis of Ki-67 score immunostaining and associations between the final diagnosis of molar subtype or GNT

The aim of testing the association between the Ki-67 score and the final diagnosis of CHM, PHM, or GNT was to obtain another tool for the diagnosis and prognosis of the disease. In the Ki-67 TMA analysis of the 108 samples, 27 samples were not evaluated for the following reasons: a) Necrosis in 11 patients; b) Scarcity of villous tissue in the section in eight patients; c) Gestational-pattern endometrium in five patients; d) Absence of marker reactivity in two patients; and e) Detachment of the fragment during histological processing in one patient, resulting in a total of 81 samples; however, three cases were inconclusive for HM and consistent with gestational nonmolar products of conception, resulting in a total of 78 participants with HMs. In the Ki-67 TMA, 57 (73 %) represented CHM patients of whom 8 had a score of 1+, 21 had a score of 2+, 27 had a score of 3+, 21 were PHM patients, one was negative for protein expression, five had a score of 1+, 12 had a score of 2+, and 4 had a score of 3+. The differences in the Ki-67 score and molar subtype between the two groups were not statistically significant. Fisher’s exact test, χ^2^ = 6.109, *p* = 0.082 (*p* > 0.05). On the basis of these results, the authors sought to test the adjusted residuals for the data via a linear-by-linear association test to evaluate the statistical trend of the Ki-67 score with respect to the final diagnosis. There was no significant association between the mole subtype and the results of Ki-67 (ordinal Chi-Square test: χ^2^ = 7.371; g.l.: *p* = 0.007) (*p* > 0.05), but there was a consistent trend toward an association between a diagnosis of CHM and a score of 3+ according to the significantly higher proportion (given the adjusted residual value of 2.3) of CHM cases (Table 1). Owing to this analysis, the authors sought a predictive marker to determine whether there was an association between Ki-67 expression and progression to GNT. Among these 78 patients with HMs, 69 were possible to follow prospectively, 17 tested negative for the marker, 2 had a score of 1+, 9 had a score of 2+ and 6 had a score of 3+; statistically, there was no association between the Ki-67 score and the development of neoplasia. (Fisher’s exact test = 1.25; *p* = 0.819; *p* < 0.05) (Table 2). A combined HER2 cell proliferation marker was also investigated to verify an association between the diagnosis of MHC, MHP, or GNT.

**Table 1:** Adjusted residuals of the final diagnostic results with those of Ki67.

**Final Diagnosis**	**Ki67 Score**			
	**0**	**+**	**++**	**+++**
Complete Mole	0.6	−1	−1.6	2.3
Partial Mole	−0.6	1	1.6	−2.3

Ordinal Chi-Square Test: χ^2^ = 7.371; g.l.: 1; *p* = 0.007.

**Table 2:** Conjugated frequencies of the postmolar gestational trophoblastic neoplasia results and Ki67 scores.

**Neoplasia**	**Ki67 Score**				**Total**
	**0**	**+**	**++**	**+++**	
Yes	‒	2	9	6	17
No	1	10	21	20	52
Total	1	12	30	26	69

Fisher’s exact test: χ^2^ = 1.25; *p* = 0.819.

### Semiquantitative analysis of HER2 immunostaining and associations between the final diagnosis of the molar subtype or GNT

In addition to the IHC Ki-67 results, the authors analyzed HER2 score immunostaining with the same objectives outlined for Ki-67. IHQ HER2 demonstrated non-evaluability in 28 (80 %) samples according to the TMA analysis. In 11 of the non-evaluable samples, the material was predominantly necrotic; in eight samples, the material contained little villous tissue; in five samples, the material was predominantly gestational-pattern endometrium; in 2 samples, the material exhibited no marker reactivity; and in the other two samples, the material detached during histological processing. Three patients were inconclusive for HM diagnosis and were diagnosed with nonmolar pregnancies; therefore, these patients were not included in the following statistical analysis. In total, 77 samples were analyzed; 18 CHM patients tested negative for the marker, 17 had a score of 1+, 11 had a score of 2+, and 9 had a score of 3+. Twelve PHM participants tested negative for the marker, 4 had a score of 1+, 5 had a score of 2+, and 1 had a score of 3+. There was no significant association between the final diagnosis and HER2 results (Fisher's exact test [χ^2^ = 4.295; *p* = 0.220] [*p* > 0.05]). The linear-by-linear association test, which was used to identify trends in the frequency distribution of the HER2 results, did not reveal a significant association between the final diagnosis and any HER2 result (Chi-Square test [χ^2^ = 2.549 g.l. *p* = 0.123] [*p* > 0.05]) (Table 3). The analysis verified that there was no evidence of an association between the final diagnosis and HER2 results for any of the comparisons. The associations between the HER2 score and progression to GTN were investigated. Sixty-seven HER2 HM patients were followed prospectively, and 17 progressed to postmolar GTN. Among these seventeen patients, five were negative for the HER2 marker, six had a score of 1+, 4 had a score of 2+, and 2 had a score of 3+. The associations of postmolar GTN with the HER2 score were not significant (Fisher’s exact test χ^2^ = 1.310; *p* = 0.773] [*p* > 0.05]) (Table 4). The TMA analysis for HER2-FISH in the previous study was used to test for sample ploidy and amplification; two patients with HER2 immunostaining scores of 2+ had amplification of the HER2 oncogene according to the FISH HER2 method. Both of these participants developed postmolar GTN. The TMA method was useful for analyzing Ki-67 in 78 cases (73 %), and HER2 analysis via TMA was possible in 77 samples (72 %).

**Table 3:** Adjusted residuals of the final diagnostic results with those of HER2.

**Final Diagnosis**	**HER2 Score**			
	0	+	+	+++
Complete Mole	−1.8	1.1	−0.3	1.4
Partial Mole	1.8	−1.1	0.3	−1.4

Ordinal Chi-Square Test (χ^2^ = 2.549; g.l.: 1; *p* = 0.123).

**Table 4:** Combined frequencies of the post-molar gestational trophoblastic neoplasia results and the HER2 score.

**Neoplasia**	**Ki67 Score**				**Total**
	0	+	++	+++	
Yes	5	6	4	2	17
No	21	13	9	7	50
Total	26	19	13	9	67

Fisher’s exact test χ^2^ = 1.310; *p* = 0.773.

## Discussion

The study investigated the cell proliferation markers Ki-67 and HER2 to better identify the two HM subtypes and identify predictive markers for GNT. Although there was no statistically significant association between Ki-67 and HER2 scores and the diagnosis of the HM subtype and GNT, there was evidence that enhanced KI-67 is related to the diagnosis of CHM, suggesting a worse prognosis. A large proportion (35 %) of HM patients with Ki-67 3+ tumors developed GTN, and two HM patients with HER2 2+ tumors had amplification of the oncogene. The data may assume predictive factors and consequently may point to targeted therapies.

Ki-67 immunostaining was observed in a large proportion of patients 78 (72 % in the present study sample), and there was a statistical tendency toward an association between CHM and a 3+ immunostaining score, reflecting the importance of further research to elucidate the association between molar subtype and Ki-67. In this work, 27 (47 %) of 57 CMs had a score of 3+, corroborating the previously reported evidence that this marker not only has a high rate of positivity in CHMs but also has diagnostic and predictive value for postmolar GTN development.^21^^,^^22^ The differential expression of molecular biomarkers may be valuable for distinguishing the subtypes of HMs and their mimics. Thus, biomarkers may be the key to refining the HM diagnosis.^23^ The importance of the biomarker Ki-67 was highlighted, and the expression of the Ki-67 protein was significantly greater in CHM than in PHM. The choice of semiquantitative analysis of Ki-67 expression in the present study sample is in line with its common use and ease of implementation in most laboratories.

In addition to its diagnostic utility, Ki-67 has prognostic value, as highlighted by the significantly increased expression of this protein in the cytotrophoblasts of samples from participants who developed trophoblastic neoplasia. There was no significant association between Ki-67 expression and postmolar GTN; however, the linear-by-linear association test revealed a trend toward an increasing frequency of CHM patients with higher expression of this marker, and consequently, a greater proportion of these patients developed GNT. Among the 17 HM patients who were evaluated and progressed to GTN, all (100 %) were immunopositive for this marker, and for 15 (88 %) of them, the immunostaining score was between 2+ and 3+. Among these patients, 5 (29 %) had CHM and 3+ immunostaining scores, demonstrating the diagnostic and prognostic importance of this marker. The difference in Ki-67 expression between patients who progressed to neoplasia and those who experienced remission of the disease was not significant, which can be attributed to the fact that the authors had fewer participants who progressed to neoplasia than did other studies that included larger samples.^12^^,^^21^^,^^22^

The importance of Ki-67 was also reported in previous work by our team, namely, a case of GTN after a tubal ectopic molar pregnancy in which p57 positivity was observed in conceptus tissue, and the cellular proliferation markers Ki-67 and p63 were highly expressed.^24^ This finding is relevant for the differential diagnosis of nonmolar hydropic abortion, which is morphologically similar to PHM. These tumor biomarkers signal the diagnosis of tubal ectopic PHM and subsequent GTN development. The current study reinforces our previous work by identifying Ki-67 as a complementary marker for the diagnosis and prognosis of HM. However, subsequent large-scale and interinstitutional trials must be carried out to demonstrate the importance of this biomarker in a more accurate diagnosis of HM. To identify other cell proliferation markers, the authors also evaluated the importance of HER2 in this study.

Studies in the literature have reported significant associations between increased HER2 protein immunopositivity in HM and progression to GTN.^25^, ^26^, ^27^ Genomic amplification by FISH HER2 occurred in two patients in our series, and there are no published reports of amplification identified via this method as a predictive factor for GTN. The two patients in whom the amplification of this oncogene was observed presented a diagnosis of CHM. These diagnoses were confirmed by the p57 marker, and the patients progressed to GTN, resulting in a worse prognosis. Notably, both of these patients had an IHC score of 2+ with the use of the HER2 marker.

There was no correlation between a HER2 score of 3+ and amplification of the oncogene, as stated in studies that investigated IHC analysis of the expression of this protein and FISH detection of HER2 in breast carcinoma, where a significant relationship was established between these two methods, mainly for scores of 1+ and 3+.^28^^,^^29^ However, these studies indicated that cases with a score of 3+ did not present amplification of the oncogene by FISH, indicating that further investigations are needed to evaluate the clinical significance of this occurrence in these cases. FISH for HER2 could play a complementary role in the detection of oncogene amplification in HM and could be used to select targeted therapy in patients who progress to postmolar GTN, as is the case for breast and colorectal carcinoma and other malignant neoplasms.^15^ HER-2 is consolidated in oncology as an important prognostic marker in several solid tumors, as breast cancer, whose treatment is especially favorable in cases where this marker is expressed and trastuzumab therapy is used.^30^ The behavior of Gestational Choriocarcinoma (GC) cells of the JEG-3 and BeWo lineages in culture was studied when these cells were exposed to a targeted drug such as Lapatinib®, which has shown promising results when used in patients with HER2-positive tumors. Similarly, trastuzumab, another anti-HER-2 monoclonal antibody, has already been shown to be effective in the treatment of choriocarcinoma.^31^ Notably, GC is the most aggressive neoplasm of the GTN and is very responsive to chemotherapy. However, a subset of patients develops an even more aggressive, metastatic form of the disease that is intrinsically resistant to traditional chemotherapy, a fact that makes it important to develop alternative or complementary therapeutic strategies to support the medical management of these patients.^29^

The HER-2 marker has also emerged as a key target in the GTN treatment, particularly in cases resistant to conventional chemotherapy. As a receptor in the epidermal growth factor family, HER-2 is often overexpressed in certain GTN subtypes, making it a promising target for biological therapies.^2^^,^^3^ The use of agents such as trastuzumab or Lapatinib®, has shown potential in improving tumor response and reducing the toxicity associated with traditional treatments. Although there are currently no clinical trials or case reports specifically addressing trastuzumab use in GTN, the experience with HER2-targeted therapies in other HER2-positive gynecologic malignancies, such as uterine serous carcinoma, demonstrates their clinical efficacy. This is exemplified by Fader et al., who reported a significant improvement in progression-free survival with the addition of trastuzumab to chemotherapy in HER2-positive uterine serous carcinoma.^32^ Furthermore, a case report by Gunduz et al. described complete remission after only two cycles of trastuzumab combined with chemotherapy in a patient with HER2-positive gastric adenocarcinoma showing choriocarcinomatous differentiation, with a marked decrease in β-hCG levels, illustrating the clinical feasibility of targeting HER2 in tumors with trophoblastic features.^33^ Additionally, in vitro studies have demonstrated that trastuzumab and Lapatinib® significantly reduce proliferation and induce apoptosis in choriocarcinoma cell lines (JEG3 and BeWo) through HER2 blockade.^34^ These findings provide mechanistic support for the potential use of HER2-targeted therapies in GTN. Collectively, these experimental and clinical observations reinforce the biological plausibility of incorporating anti-HER2 strategies in the management of HER2-positive GTN, highlighting the need for dedicated clinical studies in this specific patient population.

While this study primarily focuses on short-term prognostic markers in GTN, particularly those associated with initial treatment response and resistance, it is important to consider their potential correlation with long-term outcomes, such as overall survival and disease recurrence. Biomarkers like HER2, PD-L1, and Ki-67, which have been associated with tumor aggressiveness and chemoresistance in other malignancies, may also serve as indicators of relapse risk and long-term prognosis in GTN. Although the present study did not include an extended follow-up to assess these outcomes, previous retrospective analyses suggest that persistent overexpression of proliferative and immune-related markers could reflect a higher probability of disease recurrence or metastasis, ultimately impacting patient survival.^35^^,^^36^ Future longitudinal studies with extended follow-up are needed to validate whether the biomarker profiles observed here hold predictive value beyond the short term, helping to refine long-term management strategies for patients with GTN.

This study needs to be interpreted with caution, especially due to its small sample size, the high rate of sample exclusion, and the lack of significant associations between biomarkers and clinical outcomes, which are weaknesses that should be highlighted. The limited sample size is particularly relevant when considering subgroup analyses, such as the evaluation of HER2 amplification in GTN. In these subgroups, the reduced number of cases may have lowered the statistical power to detect meaningful associations, increasing the risk of type II errors and potentially obscuring real differences. Therefore, the results of subgroup analyses should be interpreted with additional caution, recognizing the inherent limitations related to sample size constraints. Nevertheless, the characterization of the immunoexpression of diagnostic, prognostic, and therapeutic markers in patients with GTD from the Brazilian population is important for comparison with other population groups and helps to understand possible regional differences in the outcomes of these cases. Future studies should include multicenter designs to increase the sample size and explore additional biomarkers associated not only with the diagnosis, but especially with the prognosis of this disease. The agenda for investigating new targeted therapies in GTN, a disease that affects young women of reproductive age, but can lead to death if not treated appropriately, is open.

In this study, the authors used the TMA method, which included many samples from patients with HMs in only one slide, demonstrating an economic research approach, and even with small samples and losses in this method, obtained relevant results indicating the importance of Ki-67 and HER2 markers in MH diagnosis and prognosis. The knowledge gap the authors have experienced in MH is the need for molecular diagnostic and predictive markers for the disease at the time of the histopathological examination and the precise level of malignant outcome following CHM or PHM. The present study determined the importance of Ki-67 and HER2 as biological markers that may aid in refining the diagnosis of molar subtypes and predicting patient prognosis. The molecular factors determining malignant behavior and resistance to chemotherapy need further research. More than 90 % of patients with GTN are cured by chemotherapy, including the chemotherapeutic agents etoposide and cisplatin, although some women have chemoresistant neoplasms, which represents a major therapeutic challenge.^30^^,^^31^ The present study raises the possibility of targeted HER2 therapy as an adjuvant alternative for these cases of GTN, given the substantial positivity for this protein in our cases, making it an additional treatment option for patients with more severe disease. The information obtained from these tests can also help in the selection of additional alternative targeted therapies that are better suited to the most severe and chemoresistant cases of GTN, and this possibility should be further explored in future studies.

## Abbreviations

HM, Hydatidiform Mole; CHM, Complete Hydatidiform Moles; PHM, Partial Hydatidiform Mole; TMA, Tissue Microarray; GTN, Trophoblastic Neoplasia; GTD, Gestational Trophoblastic Disease; FIGO2021, ‘Federation Internationale de Gynecologie et Obstétrique’ 2021; GC, Gestational Choriocarcinoma.

## Author statement

This work was conducted according to the STARD guidelines.

## Declaration of generative AI and AI-assisted technologies in the writing process

None to declare.

## Funding

This study did not receive any specific grant from funding agencies in the public, commercial, or not-for-profit sector.

## CRediT authorship contribution statement

**Consuelo Lozoya López:** Conceptualization, Methodology, Investigation, Writing – original draft, Visualization, Project administration. **Nathália Silva Carlos Oliveira:** Formal analysis, Writing – original draft, Writing – review & editing, Visualization. **Fabiana Resende Rodrigues:** Validation, Investigation, Resources, Writing – review & editing, Supervision, Project administration. **Ana Luisa Figueira Gouvêa:** Writing – review & editing, Supervision. **Karin Soares Gonçalves Cunha:** Writing – original draft. **Ana Carolina Brito:** Methodology, Visualization. **Antonio Braga:** Resources, Data curation, Writing – original draft, Writing – review & editing. **Vânia Gloria Silami Lopes:** Project administration, Supervision.

## Declaration of competing interest

The authors declare no conflicts of interest.
